# New Stability-Indicating RP-HPLC Method for Determination of Diclofenac Potassium and Metaxalone from their Combined Dosage Form

**DOI:** 10.3797/scipharm.1109-16

**Published:** 2011-12-05

**Authors:** Sagar Suman Panda, Debasis Patanaik, Bera V. V. Ravi Kumar

**Affiliations:** Department of Pharmaceutical Analysis and Quality Assurance, Roland Institute of Pharmaceutical Sciences, Khodasingi, 760010, Berhampur (Orissa), India

**Keywords:** Diclofenac potassium, Metaxalone, Forced degradation study, HPLC

## Abstract

A simple, precise and accurate isocratic RP-HPLC stability-indicating assay method has been developed to determine diclofenac potassium and metaxalone in their combined dosage forms. Isocratic separation was achieved on a Hibar-C_18_, Lichrosphere-100^®^ (250 mm × 4.6 mm i.d., particle size 5 μm) column at room temperature in isocratic mode, the mobile phase consists of methanol: water (80:20, v/v) at a flow rate of 1.0 ml/min, the injection volume was 20 μl and UV detection was carried out at 280nm. The drug was subjected to acid and alkali hydrolysis, oxidation, photolysis and heat as stress conditions. The method was validated for specificity, linearity, precision, accuracy, robustness and system suitability. The method was linear in the drug concentration range of 2.5–30 μg/ml and 20–240 μg/ml for diclofenac potassium and metaxalone, respectively. The precision (RSD) of six samples was 0.83 and 0.93% for repeatability, and the intermediate precision (RSD) among six-sample preparation was 1.63 and 0.49% for diclofenac potassium and metaxalone, respectively. The mean recoveries were between 100.99–102.58% and 99.97–100.01% for diclofenac potassium and metaxalone, respectively. The proposed method can be used successfully for routine analysis of the drug in bulk and combined pharmaceutical dosage forms.

## Introduction

**Fig. 1:**
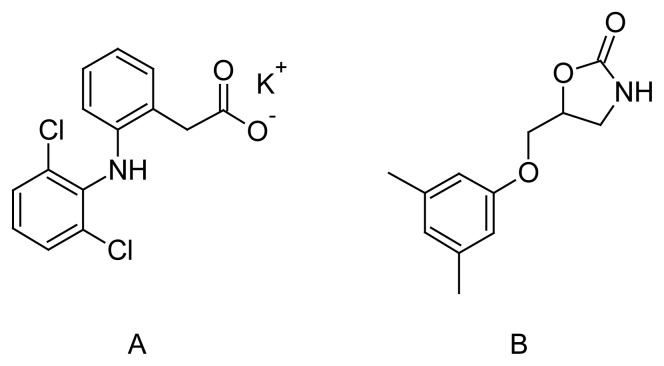
Chemical structures of (A) Diclofenac potassium and (B)Metaxalone

Diclofenac Potassium (DIC), potassium {2-[(2,6-dichlorophenyl)amino]phenyl}acetate (Figure 1A), is a non-steroidal anti-inflammatory drug (NSAID) that exhibits anti-inflammatory, analgesic, and antipyretic activities [1]. Metaxalone (MET), 5-[(3,5-dimethyl-phenoxy)methyl]-1,3-oxazolidin-2-one (Figure 1B), is a skeletal muscle relaxant [2].

Literature survey shows that no stability-indicating RP-HPLC method has been reported so far for the simultaneous estimation of both the drugs using mobile phase of methanol: water (80:20%, v/v). Some of the reported methods for DIC include UV-spectroscopic methods [3–6], RP-HPLC methods [7–10] and LC-MS method [11]. For MET the reported methods are UV-Spectroscopic method [12], RP-HPLC method [13, 14] and LC-MS method [15]. Only one RP-HPLC method has been reported so far for simultaneous estimation of both the drugs [16], but the reported method is not stability-indicating in nature. The objective of this work was to develop a novel, simple, precise and rapid RP-HPLC method which can be used as a stability-indicating assay for combination drug product of DIC and MET.

To establish the stability-indicating nature of the method, forced degradation of drug substances and drug product was performed under stress conditions (acid, alkali, oxidation, thermal and photolysis), and stressed samples were analyzed by the proposed method. The method was also validated according to ICH guideline requirements [17].

## Experimental

### Chemicals and Reagents

Analytical Grade DIC and MET (purity > 99.5%) were procured from Sun Pharmaceuticals Industries Ltd., India. Methanol (Merck Ltd., Mumbai, India) was of HPLC grade. Analytical grade sodium hydroxide, hydrochloric acid and hydrogen peroxide were procured from S.D. Fine Chem. Ltd., Mumbai, India. The water for HPLC was obtained by using TKA Water Purification System, Germany. The combined tablet formulation (Flexura-D, Sun Pharmaceuticals Industries Ltd., India) containing 50 mg of DIC and 400mg of MET was purchased from the local market.

### HPLC Instrumentation and chromatographic conditions

Quantitative HPLC was performed on a binary gradient HPLC with Shimadzu LC-10AT and LC-10AT VP Series HPLC pumps, with a 20 μl sample injection loop (manual) and SPD 10A VP series UV Visible detector. The output signal was monitored and integrated using Shimadzu Class-VP Version 6.12 SP1 Software. A Hibar-C_18_, Lichrosphere-100^®^ (250 mm × 4.6 mm i.d., particle size 5 μm) was used for separation. Chromatographic analysis was carried out at ambient temperature on the column using the methanol: water (80:20, v/v) as mobile phase at a flow rate of 1.0 ml/min in isocratic mode. The column back pressure was found to be 164 kgf/cm^2^. The mobile phase utilizes no special preparation technique except filtration using 0.45μm filter paper. Afterward both the methanol and water were ultrasonicated (Enertech, India) up to 20 minutes for degassing prior to use. The UV detection wavelength was 280 nm. Water bath (Thermolab, India) and UV Chamber (Jain Scientific Glass Works, Ambala, India) were used for forced degradation study of the drugs. Analytical balance, Model-GR-202 (AND Instrument India Pvt. Ltd., Gurgaon, India) of sensitivity 0.1 mg was used to weigh the chemicals and reagents.

### Preparation of Standard and Sample Solution

Standard stock solutions for both the drugs were prepared separately by dissolving 25 mg of the drugs in methanol up to 25 ml. The volumetric flasks having 10 ml of methanol were ultrasonicated for 5 minutes. Finally, the volumes were made up to the mark with methanol, which gave 1000μg/ml solutions. From this a mixed standard stock solution was prepared so that the drugs DIC and MET were in the ratio equal to that of the marketed formulation (1:8) available. Working standard solutions of DIC and MET were prepared in the concentration ratio of 1:8.

Twenty tablets were weighed accurately and finely powdered. A quantity of tablet powder equivalent to 25 mg of DIC and 200 mg of MET was accurately weighed and transferred into a 25 ml volumetric flask, containing 10 ml of mobile phase and ultrasonicated for 20 min; the volume was made up to the mark and mixed well. The solution was filtered through a 0.2 μm filter to ensure the absence of particulate matter. The filtered solution was appropriately diluted with the mobile phase for analysis as already described. The amount of drug present in the sample solution was calculated by using the calibration curves.

### Method validation

#### Specificity

The specificity of the method was determined by checking the interference of any of the possible degradation products generated during the forced degradation study of the drugs. The forced degradation of the drug was carried out with 0.1 N HCl, 0.1 N NaOH, 3% v/v hydrogen peroxide, heat (80°C) and photolysis (365 nm) for determining the stability nature of the drugs. The degradation samples were prepared by taking suitable aliquots of the drug and drug product solutions, and then undertaking the respective stress testing procedures for each solution. After the fixed time period the treated test solutions were diluted up to the mark with mobile phase. For every stress condition three solutions were prepared as 10 μg/ml of DIC, 80 μg /ml of MET and drug product solution containing 10 μg/ml of DIC along with 80 μg/ml of MET. The specific stress conditions are described as follows.

Acidic degradation condition:

Acidic degradation was carried out by adding 2 ml of 0.1N HCl, and after 45 minutes neutralizing the mixture by adding 0.1N NaOH.

Alkali degradation condition.

Alkali degradation was carried out by adding 2 ml of 0.1N NaOH, and after 45 minutes neutralizing the mixture by adding 0.1N HCl.

Oxidative degradation condition:

Oxidative degradation was performed by exposing the drug to 2 ml of 3% (v/v) H_2_O_2_ for 45 minutes.

Thermal degradation condition:

Thermal degradation was performed by heating the drug content at 80ºC on a thermostatically controlled water bath for 45 minutes.

Photolytic degradation condition:

Photolytic degradation was carried out by exposing the drug content to UV light (365nm) inside a UV chamber for 180 minutes.

#### Linearity

Eight point calibration curves were obtained in a concentration range from 2.5 to 30 μg/ml for DIC and 20 to 240 μg/ml for MET. Calibration curves were plotted by taking the average peak area (n=3) on y-axis and concentration (μg /ml) on x-axis for DIC and MET separately.

#### Precision

The repeatability (intra-day precision) of the method was ascertained from the peak areas obtained by actual determination of six replicates of a fixed amount of drug. For intermediate precision (inter-day precision) of the method the above same procedure was carried out by a different analyst on a different day under similar experimental conditions. The percent RSD values were calculated for each type of precision study.

#### Accuracy

To check the accuracy of the proposed method, recovery studies were carried out at 80, 100 and 120% of the test concentration. The recovery study was performed three times at each level. The amount of DIC and MET present in the sample were calculated using the calibration curves.

#### Robustness

The robustness of the method was studied by very deliberately changing the composition of mobile phase and by determining the solution stability of the sample solution at 25 ± 2°C for 24 h. The method was also evaluated for different system suitability parameters like Retention time, Theoretical plate, Asymmetry and Resolution.

#### Limit of detection and Limit of quantitation

The limit of detection and limit of quantitation were separately determined based on the Signal to Noise ratio. For limit of detection the S/N ratio was taken as 3:1. For limit of quantitation the S/N ratio was taken as 10:1.

## Results and Discussion

### Optimization of the Chromatographic conditions

**Fig. 2:**
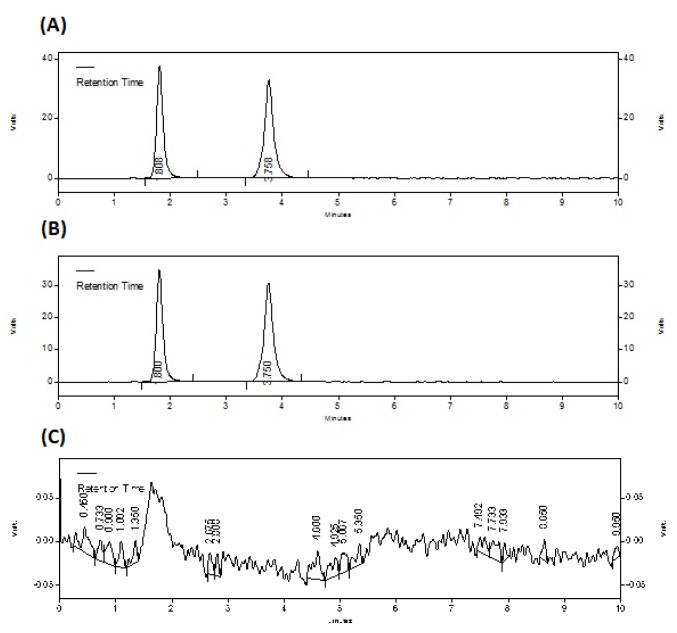
Chromatograms of (A) standard drugs, (B) drugs in combined dosage form, (C) blank mobile phase run

Optimization of mobile phase was carried out based on resolution, tailing factor and theoretical plates obtained for DIC and MET. During the trial runs both the drugs were tested with different mobile phase compositions like acetonitrile: water, acetonitrile: 0.01M tetra butyl ammonium hydrogen sulphate, methanol: water and methanol: 0.01M tetra butyl ammonium hydrogen sulphate at various compositions and flow rates. The mobile phase consisting of methanol: water (80:20, v/v) at a flow rate of 1.0 ml/min was selected which gave sharp, well-resolved peaks for DIC and MET. The retention times for DIC and MET were 1.808 and 3.758 minutes, respectively. The asymmetry for DIC and MET were 1.21 and 1.13, respectively. UV spectra of DIC and MET in mobile phase ratio showed that both the drugs absorbed UV radiations appreciably at 280 nm, so the same was selected as the detection wavelength. The separation was carried out at room temperature. Fig. 2 represents the chromatograms of standard drugs, drugs in combined tablet formulation and blank mobile phase run, respectively.

### Specificity

Specificity of the method was ascertained by checking any interference due to excipients or degradation products. No extra peaks were obtained either from the excipients used in the drug product or from the stress conditions applied on the drugs and drug product. Hence, there was no interference with the drug peaks. In acidic (45.12%), alkali (24.68%) and oxidation (16.97%) conditions DIC shows some significant degradation than compared to thermal (4.01%) and photolytic (3.48%) stress conditions. But the drug MET was found to be relatively more stable with very less degradation (1.05–4.39%) in all the applied stress conditions than compared to DIC. Fig. 3 represents the chromatograms of untreated drugs; oxidation degraded drugs and thermally degraded drugs with drug products, respectively.

### Linearity

The two eight point calibration curves were found to be linear over a concentration range of 2.5–30 μg/ml and 20–240 μg/ml for DIC and MET, respectively. The linear regression equations were *y* = 31587 × − 1914.6 and *y* = 4656.5 × + 6788.5 with correlation coefficients 0.9992 and 0.9994 for DIC and MET, respectively.

### Precision

The method was found to be precise as the % RSD values for repeatability and intermediate precision studies were well below 2%, confirming that the method was precise. The results are shown in Table 1.

### Accuracy

Accuracy of the method was determined by recoveries of DIC and MET by standard addition methods. The average recoveries were 100.99–102.58% for DIC and 99.97–100.01% for MET, respectively. The values show high levels of accuracy of the method. The result of accuracy study is shown in Table 2.

### Robustness

Robustness of the method was ascertained by deliberately changing the mobile phase composition to methanol: water (79:21, v/v) by increasing percentage of water, the retention time for DIC and MET were observed to be 1.850 and 3.767 minutes, respectively. Similarly, when the percentage of methanol was increased in methanol: water (81:19, v/v) the retention time for DIC and MET were found to be 1.858 and 3.758 minutes, respectively. The solution stability study shows that DIC and MET solutions were stable for 24 h at ambient conditions without any significant degradation of the analyte. The results for system suitability parameters were also found to be satisfactory. The obtained results for robustness study and system suitability parameters are shown in Table 3.

### Limit of detection and Limit of quantitation

The limit of detection values for DIC and MET were 0.026 μg/ml and 0.03 μg/ml, respectively. The limit of quantification values for DIC and MET were 0.088 μg/ml and 0.1 μg/ml, respectively.

### Analysis of combined tablet dosage form

The developed method was applied for determination of DIC and MET in their combined dosage form. The higher percentage of recovery and non interference of the formulation excipients in retention time of the drugs show the selectivity of the method for estimation of both drugs in their combined dosage forms. The result of assay (n=3) for both drugs yielded 100.96% (S.D.=±0.91) and 101.09% (S.D.=±0.78) for DIC and MET, respectively, from the combined tablet dosage form.

## Conclusion

A validated stability-indicating RP-HPLC method has been developed for determination of DIC and MET in their combined tablet dosage form. The results obtained by the stress degradation conditions of the drugs show that the method is specific and stability-indicating. The drug MET was found to be more stable to degradation under different stress conditions than compared to DIC. The method was found to be simple, accurate, precise and sensitive. The method was successfully applied for the determination of both drugs in combined tablet dosage form. In the future, this method may be applied for routine analysis of both the drugs in API, formulations, dissolution studies, bioavailability and pharmacokinetic studies.

## Figures and Tables

**Fig. 3A:**
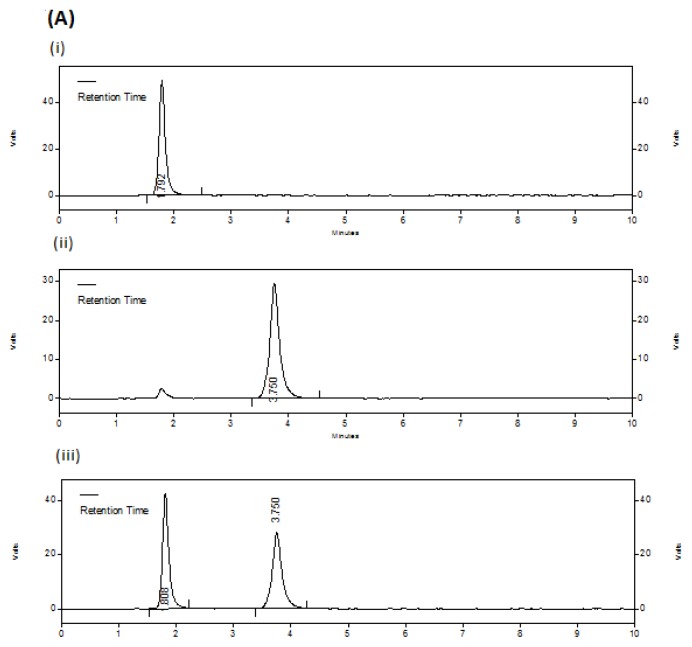
Chromatograms of untreated drugs Each chromatogram represents (i) DIC (10 μg/ml), (ii) MET (80 μg/ml), (iii) combined dosage form with DIC (10 μg/ml) and MET (80 μg/ml)

**Fig. 3B:**
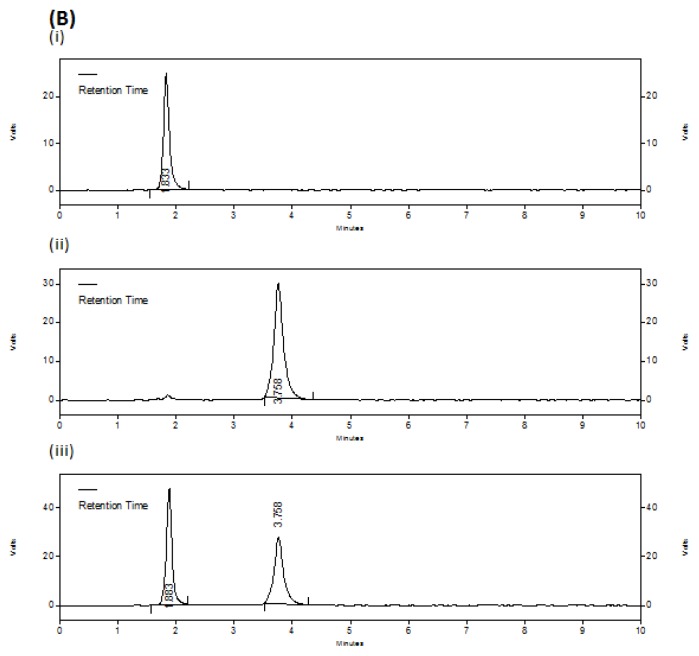
Chromatograms of oxidation degraded drugs Each chromatogram represents (i) DIC (10 μg/ml), (ii) MET (80 μg/ml), (iii) combined dosage form with DIC (10 μg/ml) and MET (80 μg/ml)

**Fig. 3C:**
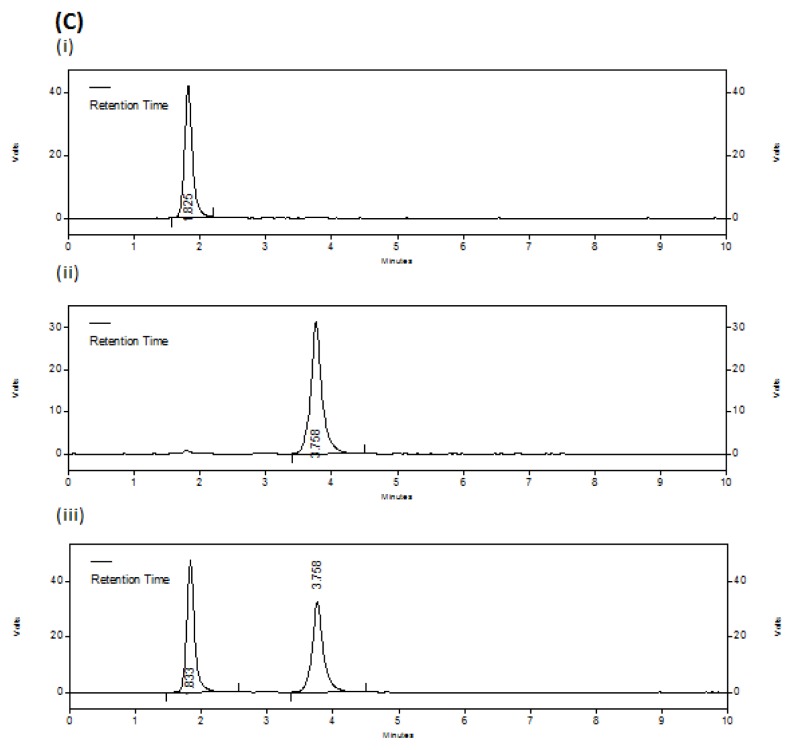
Chromatograms of thermally degraded drugs Each chromatogram represents (i) DIC (10 μg/ml), (ii) MET (80 μg/ml), (iii) combined dosage form with DIC (10 μg/ml) and MET (80 μg/ml)

**Tab. 1:** Precision of the method

	Concentration (μg/ml)		Peak Area^a^ ±Standard Deviation		% RSD	
	
Precision Type	DIC	MET	DIC	MET	DIC	MET
Repeatability (Intraday, n=6)	10	80	305213.16 ± 2533.09	374156.16 ± 3496.85	0.83	0.93
Intermediate precision (Interday, n=6)	10	80	303097.05 ± 4959.05	375515 ± 1836.54	1.63	0.49

Mean of six determinations.

**Tab. 2:** Accuracy of the method

Recovery Type (%)	Amount added (μg/ml)		% Recovery^a^ ± Standard Deviation		% RSD	

	DIC	MET	DIC	MET	DIC	MET
80	8	64	100.99 ± 0.99	100.01 ± 0.18	0.98	0.18
100	10	80	101.46 ± 0.32	100.01 ± 0.33	0.32	0.33
120	12	96	102.58 ± 0.28	99.97 ± 0.03	0.27	0.03

a Mean of three determinations.

**Tab. 3:** Robustness of the method

	Standard condition	Modified conditions	Retention Time, minutes		Resolution
				
			DIC	MET	
**Mobile phase ratio**	80:20 (v/v)	79:21 (v/v)	1.850	3.767	4.79
		81:19 (v/v)	1.858	3.758	4.75

	Initial time	Final time	% Recovery	
			
			DIC	MET
**Solution stability**	0 h	24 h	99.5	100.5

	System Suitability	
	
	MET	DIC
**Retention time (min)**	1.808	3.758
**Theoretical plates**	2153	2758
**Asymmetry**	1.21	1.13
**Resolution**	4.87	–

## References

[1] Sweetman S (2009). Analgesics Anti-inflammatory Drugs and Antipyretics. Martindale-The Complete Drug Reference.

[2] Sweetman S (2009). Muscle Relaxants. Martindale-The Complete Drug Reference.

[3] Umarkar AR, Bagad YM, Bhurat MR, Kawatikwar PS (2011). Absorption correction method for estimation of thiocolchicoside and diclofenac potassium in combined capsule dosage form. Int J Pharm Sci.

[4] Umarkar AR, Mehta SA, Chaple DR, Thote LT (2011). Simultaneous estimation of famotidine and diclofenac potassium by UV spectrophotometer using multicomponent method. J Pharm Res.

[5] Vanparia DJ, Shah SA, Marolia BP, Bodiwala KB, Patadia RK (2011). Spectrophotometric methods for simultaneous estimation of thiocolchicoside and diclofenac potassium in their combined dosage form. Asian J Res Chem.

[6] Sanjay Kumar R, Karthikeyan C, Moorthy NSHN, Trivedi P (2006). New spectrophotometric methods applied to the simultaneous determination of diclofenac potassium and tizanidine. Indian J Pharm Sci.

[7] Rele RV, Parab JM, Mhatre VV, Warkar CB (2011). Simultaneous rp-hplc determination of diclofenac potassium and famotidine in pharmaceutical preparations. Res J Pharmacy Technol.

[8] Gowramma B, Muralidharan S, Meyyanathan SN, Suresh B (2010). A validated rp-hplc method for simultaneous estimation of paracetamol and diclofenac potassium in pharmaceutical formulation. Int J Chem Tech Res.

[9] Rathnam MV, Singh RR (2010). Simultaneous rp-hplc determination of camylofin dihydrochloride and diclofenac potassium in pharmaceutical preparations. Pharm Anal Acta.

[10] Elkady E (2010). Simultaneous determination of diclofenac potassium and methocarbamolin ternary mixture with guaifenesin by reverse phase liquid chromatography. Talanta.

[11] Prakash TB, Sri KV, Krishna SR (2009). Determination of diclofenac potassium in human plasma by lc-ms. Asian J Chem.

[12] Priyadarshini J, Saraswathi D, Ajithadas A, Jerad Suresh A (2011). Hydrotropic solubilisation technique of metaxalone by area under curve and second order derivative spectroscopy. Res J Pharmacy Technol.

[13] Nagavalli D, Sankar ASK, Anandkumar K, Vetrichelvan T, Nalaji M (2010). Estimation of metaxalone in bulk and in tablet dosage form by rp-hplc. Res J Pharmacy Technol.

[14] Cacae J, Reilly EE, Amann A (2004). Comparison of dissolution of metaxalone tablets (skelaxin) using USP apparatus 2 and 3. AAPS Pharm Sci Tech.

[15] Nirogi RV, Kandikere VN, Shukla M, Mudigonda K, Shrivastava W, Datla PV (2006). Quantification of metaxalone in human plasma by liquid chromatography coupled to tandem mass spectrometry. J Anal Toxicol.

[16] Gabhane KB (2010). Simultaneous estimation of metaxalone and diclofenac potassium in combined dosage form by high performance liquid chromatography method. Int J Chem Sci.

[17] International Conference on Harmonization (ICH) (2005). ICH Harmonized Tripartite Guideline. Topic Q2(R1). Validation of Analytical Procedures: Text and Methodology.

